# Up-regulation of Interleukin-21 Contributes to Liver Pathology of Schistosomiasis by Driving GC Immune Responses and Activating HSCs in Mice

**DOI:** 10.1038/s41598-017-16783-7

**Published:** 2017-11-30

**Authors:** Yanyan Wang, Cai Lin, Yun Cao, Zhongliang Duan, Zhixun Guan, Jing Xu, Xing-Quan Zhu, Chaoming Xia

**Affiliations:** 1 Department of Parasitology, Medical College of Soochow University, Suzhou, Jiangsu Province China; 2 State Key Laboratory of Veterinary Etiological Biology, Key Laboratory of Veterinary Parasitology of Gansu Province, Lanzhou Veterinary Research Institute, Chinese Academy of Agricultural Sciences, Lanzhou, Gansu Province China

## Abstract

The pathology of schistosome egg-induced liver granuloma, fibrosis and eventually liver scarring is complicated. CD4^+^ helper T (Th) cells play critical roles in both host humoral immunity and cellular immunity against parasitic infection and immunopathology in schistosomiasis. Follicular helper T (Tfh) cells are another specialized subset of Th cells and involved in infectious diseases. However, the immune regulatory mechanism of Tfh cells in severe liver pathology of schistosomiasis is still poorly understood. In this study, using a *S*. *japonicum*-infected mouse model, we studied the dynamics and effects of Tfh cells *in vivo* and demonstrated that Tfh phenotype molecules ICOS, PD-1 and functional factor IL-21 were positively correlated with disease development by flow cytometry. Meanwhile, our results also showed that Tfh cells enriched in splenic germinal center (GC) and promoted B cells producing IgM with the progress of hepatic immunopathology by B-T co-culture experiments. More importantly, our data indicated that IL-21 contributed to the formation and development of hepatic egg granuloma and subsequent fibrosis by driving GC responses and activating HSCs by immunohistochemical detection and blocking assay *in vitro*. Our findings contribute to the better understanding of the immunopathogenesis of schistosomiasis and have implications for therapeutic intervention of hepatic fibrotic diseases.

## Introduction

Schistosomiasis remains an important chronic infectious disease in the tropical and subtropical areas of the world, which not only has a higher morbidity but also has a serious threat to human health and social development. Following *Schistosoma mansoni* and *Schistosoma japonicum* infections, the formation of host tissue lesion (granuloma, fibrosis and eventually scarring), especially in the liver, is predominantly caused by the immunopathologic changes in response to *Schistosoma* eggs. Thus, we need to understand the mechanism of host humoral and cellular immune responses to prevent hepatic fibrogenesis in schistosome infection.

Differentiation of naïve CD4^+^ T cells into distinct T helper subsets is important for high level immune responses against various pathogens and parasites^1–3^. It is well known that CD4^+^ helper cells, including Th1, Th2, Th17 and Treg cells have also been implicated in schistosome ova-induced liver granulomatous inflammation and fibrosis. Follicular helper T (Tfh) cells is another specialized subset of Th cells and characterized by constructive expression of membrane surface molecules CXCR5 and high expression of inducible costimulator (ICOS), programmed death 1(PD-1), signaling lymphocyte activation molecule-associated protein(SAP), transcription factor Bcl-6 and functional factor IL21. A previous study showed that down-regulation of Tfh cells development or function would result in immune deficiencies^4^. In addition, several studies have focused on the contribution of Tfh cells to autoimmune and chronic inflammatory disease^5–7^. Although recent studies demonstrated that Tfh cells promote liver granulomas inflammation in mice infected with *S*. *mansoni and S*. *japonicum* ^7,8^, however, whether Tfh cells play an important role in hepatic fibrogenesis in schistosomiasisis is unknown.

Germinal centers (GC) are specialized immune structures, developed within B cell follicular of secondary lymphoid tissues, played an important role in the strength of humoral immune response. More recently, studies suggested that follicular Tfh cells were observed in the GC and have been implicated in GC formation^9–11^. In addition, follicular Tfh cells provide potent help to antigen-specific naive B cells compared to Th1 and Th2 cells, which contributes to the generation of plasma cells and memory B cells from antigen-selected high-affinity GC B cells, consequently ensuring long-term humoral immune response^12–16^. The production of IL-21 is primarily secreted by Tfh cells, and IL-21 is capable of controlling Tfh cells and Th17 cells responses^17^. A recent study suggested that increased levels of IL-21 in serum were associated with disease severity in patients with chronic hepatitis B^18^. In addition to providing help to B cells, it is not yet clear whether IL-21 of Tfh cells have other functions, and whether is involved in the development of liver pathology in schistosomiasis.

Here, using the *S*. *japonicum*-infected mouse model, we studied Tfh development and function in host immunopathogenesis by examining the basic immunology characteristics of Tfh cells during *S*. *japonicum* infection. We identified an important cytokine IL-21 of Tfh cells which was closely related with hepatic fibrosis progression. Except for providing help to regulate B cells response, IL-21 was one of the key factors in driving HSCs to produce more hyaluronic acid (HA) to promote liver pathology. Therefore, the elucidation of roles of Tfh cells and IL-21 may provide new insights into the immunopathology of liver fibrosis.

## Results

### Up-regulation of phenotyptic molecules of Tfh cells positively correlates with hepatic fibrosis progression in murine schistosomiasis

**Figure 1:**
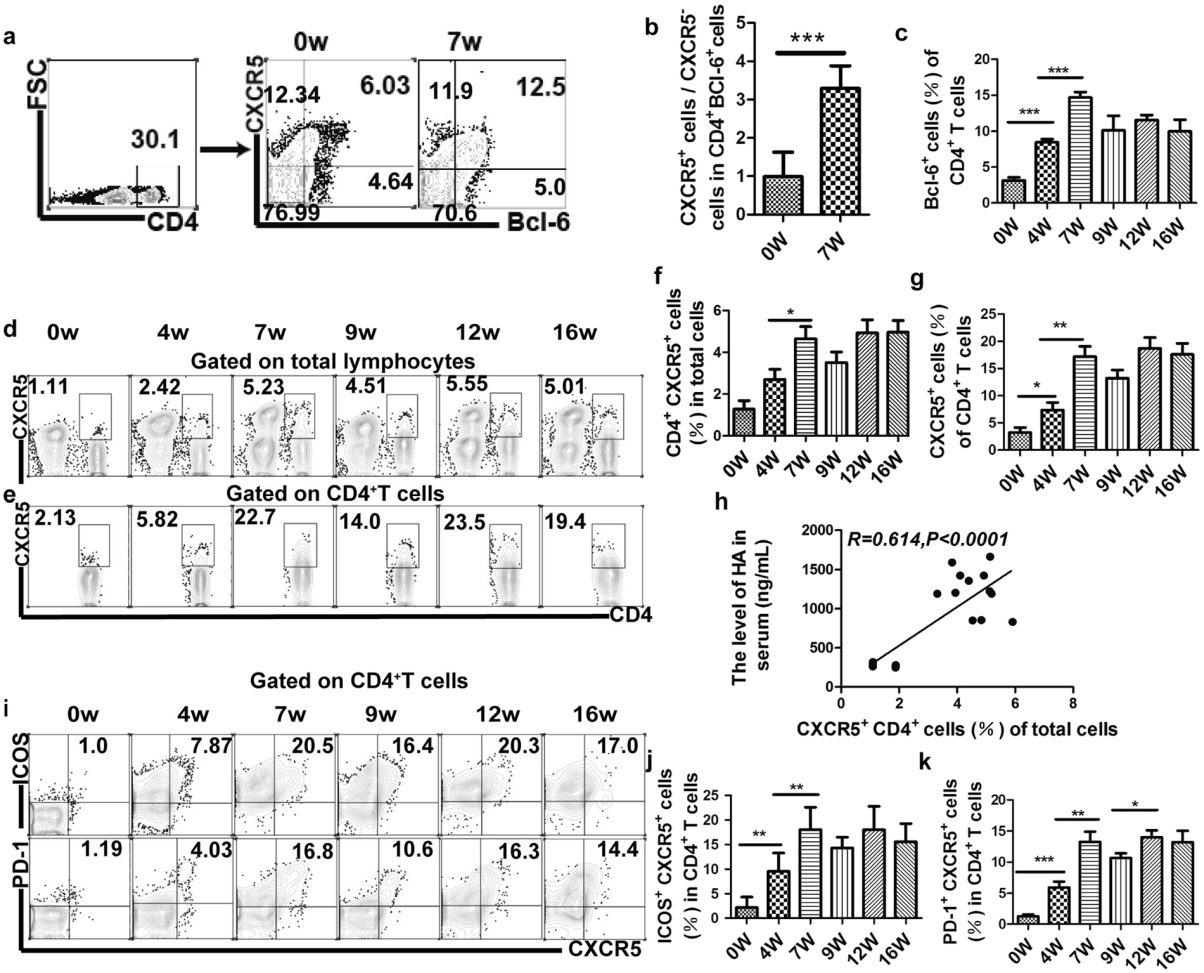
Dynamics of CD4^+^ T follicular helper cell in mice infected with *Schistosoma japonicum*. The FVB mice were infected with 15 cercariae of *S*. *japonicum*. (**a**) Normal and infected mice (7 weeks post-infection) were sacrificed, the splenic cells were directly stained with CD4-FITC, CXCR5-PE antibody and subsequently intracellular stained with Bcl-6-PE. Numbers are indicated in the upper right of each chart which represent the percentage of Bcl-6^+^CXCR5^+^ cells within the CD4^+^ T cells population; (**b**) Pooled data are from two independent experiments, values are given as mean+/− SD (n = 6), *P < 0.05; ***P < 0.001 (Student’s *t*-test); (**c**) Five mice per group were sacrificed at 0, 4, 7, 9, 12, 16 weeks post-infection, pooled data are from two independent experiments, values are given as mean+/− SD (n = 6), ***P < 0.001 (Student’s *t*-test); Splenic cells were harvested from normal and infected mice (4, 7, 9, 12, 16 weeks post-infection), surface stained with CD4-FITC, CXCR5-PE, ICOS-PE-Cy5.5, PD-1-APC antibody. FACS dot plots shows the proportions of CXCR5^+^CD4^+^ positive cells within total lymphocytes population (**d**) or CD4^+^ T cell population (**e**); (**f**), (**g**) The data shows mean+/− SD (n = 6) from two independent experiments, *P < 0.05; **P < 0.01 (Student’s *t*-test); (**h**) The percentage of CXCR5^+^CD4^+^ Tfh cells in total lymphocytes population is correlated positively with the level of HA in serum; (**i**,**j**,**k**) The proportion of ICOS^+^CXCR5^+^ and PD-1^+^CXCR5^+^ Tfh cells in CD4^+^ population are expressed as the mean+/− SD of 6 mice from two independent experiments, *P < 0.05; **P < 0.01 ***P < 0.001 (Student’s *t*-test).

Mice were infected with *S*. *japonicum* and euthanized at 0 (before infection), 4 (early stage), 7 (acute stage), 9 (acute stage), 12 (chronic stage) and 16 (advanced stage) weeks post-infection. Splenocytes were harvested, and the levels of Tfh specific phenotypic molecules were assayed with flow cytometry. Transcription factor Bcl-6 as a critical regulator of Tfh cell differentiation and expression of Bcl-6 is sufficient to induce Tfh development and function^9,19^. As shownin Fig. 1a and b, CXCR5^+^ cells was induced 3 to 4-fold higher than CXCR5^−^cells on Bcl-6^+^CD4^+^ cells in infected mouse, while normal mouse was found to increase 1to 2-fold. More importantly, Bcl-6 was up-regulated significantly at early stage and peaked at acute stage (Fig. 1c). When the disease progressed from the chronic to the advanced stage, the expression level of Bcl-6 still kept relatively higher level compared to that of before infection. Taken together, our Bcl-6 data indicated that Bcl-6 instructed Tfh development to commence early during *S*. *japonicum* infection.

**Figure 2:**
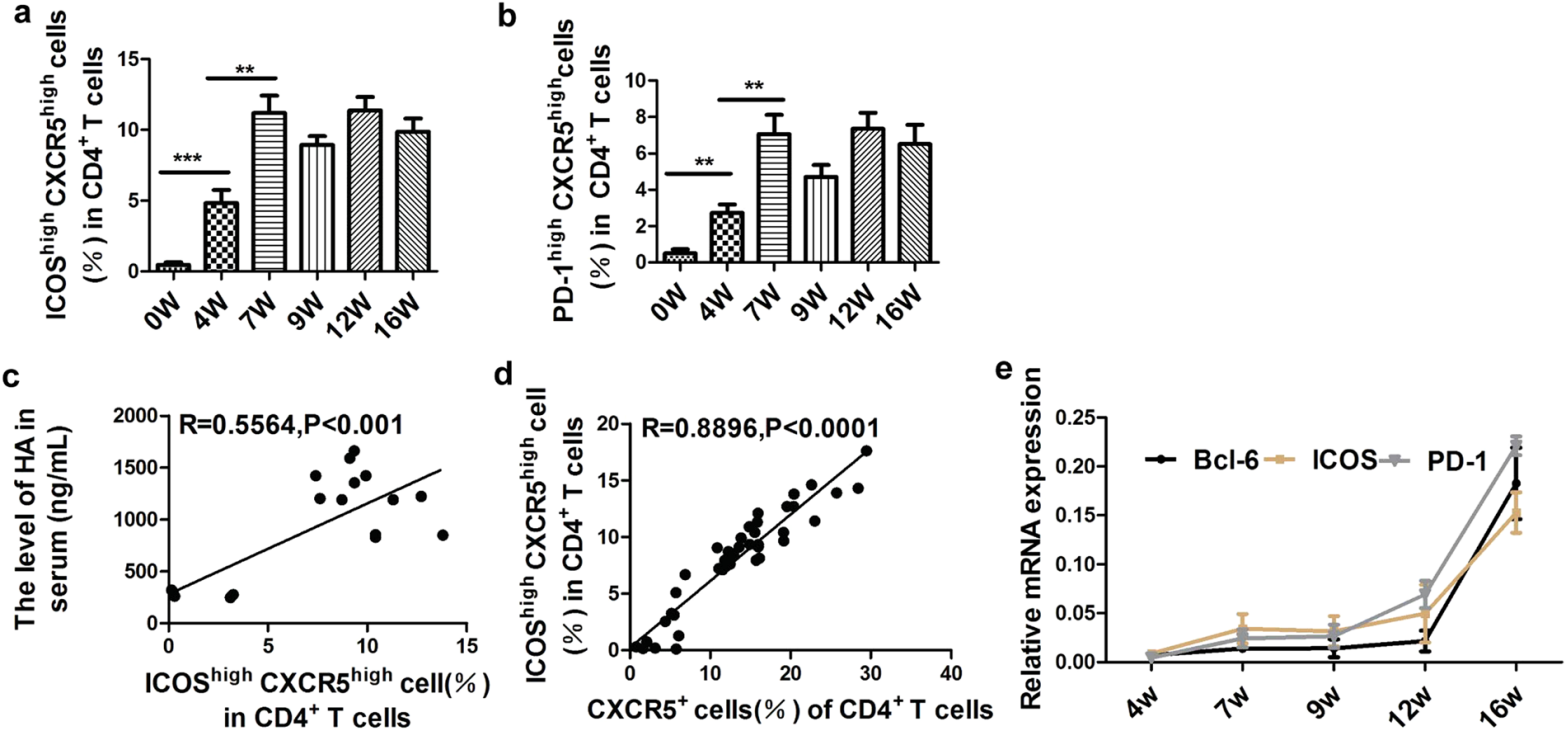
Dynamics of CD4^+^ T follicular helper cell in mice infected with *Schistosoma japonicum*. (**a**,**b**) The expression levels of ICOS^high^CXCR5^high^ and PD-1^high^CXCR5^high^ in CD4^+^ T population are expressed as the mean+/− SD of 6 mice from two independent experiments, *P < 0.05; **P < 0.01 ***P < 0.001 (Student’s *t*-test); (**c**,**d**) ICOS^high^ Tfh cells increasing was correlated positively with the level of HA in serum and Tfh cells proliferation; (**e**) Tfh cells were sorted in spleen from normal and infected mice (4, 7, 9, 12, 16 weeks post-infection) and Tfh typical gene was analyzed using 2^−ΔΔCT^ method and the housekeeping genes was β-actin. Three mice per group, data are shown as mean +/− SD from a single experiments representative of two repeated experiments.

To elucidate the mechanism of liver pathology in schistosomiasis, here we investigated the dynamics of Tfh cell proliferation and phenotypes in mice infected with *S*. *japonicum*. As shown in Fig. 1d–g, the percentages of CXCR5^+^ CD4^+^ Tfh cells in the splenic lymphoid cells were elevated at 4 weeks post-infection and reached a plateau by 12 weeks post-infection. Meanwhile, the proportion of CXCR5^+^ CD4^+^ Tfh cells in splenic CD4^+^ T cells kept increasing rapidly from week 0 through week 7 after infection and decreased at 9 weeks post-infection, but continues to increase at 12 weeks post-infection. The level of HA is an important marker of liver fibrosis degree^20,21^. To further investigate the potential correlation between increased Tfh cells and the disease severity, we examined the HA titers in mice following *S*. *japonicum* infection (Supplementary Fig. 1) and analyzed the association between the HA titers and the proportion of Tfh cells in the hepatic fibrosis progression. The results showed that increased Tfh cells were positively correlated with the level of HA in mice infected with *S*. *japonicum* (Fig. 1h and Supplementary Fig. 1). Further polarization and function of Tfh cells are associated with high expression of ICOS, PD-1,CD40L, OX40. Our results showed that the proportion of ICOS^+^ Tfh cells and PD-1^+^ Tfh cells were also increased much more quickly during the first seven weeks post-infection and kept a higher level subsequently (Fig. 1i–k). Meanwhile, a similar trend of ICOS^high^ CXCR5^high^ Tfh cells and PD-1^high^ CXCR5^high^ Tfh were observed (Fig. 2a and b). Moreover, there was a significant correlation between ICOS^high^, PD-1^high^ Tfh cells and HA levels (Fig. 2c and Supplementary Fig. 1) or CXCR5^+^ Tfh cells (Fig. 2d and Supplementary Fig. 1). However, it is surprising that the mRNA expression levels of typical genes of Tfh cells were significantly up-regulated by RT-PCR at advanced stage (16 weeks post-infection) compared to their protein expression levels by flow cytometry (Fig. 2e).

### Increased Tfh-type cytokines IL-21 positively correlates with hepatic fibrosis progression in murine schistosomiasis

**Figure 3:**
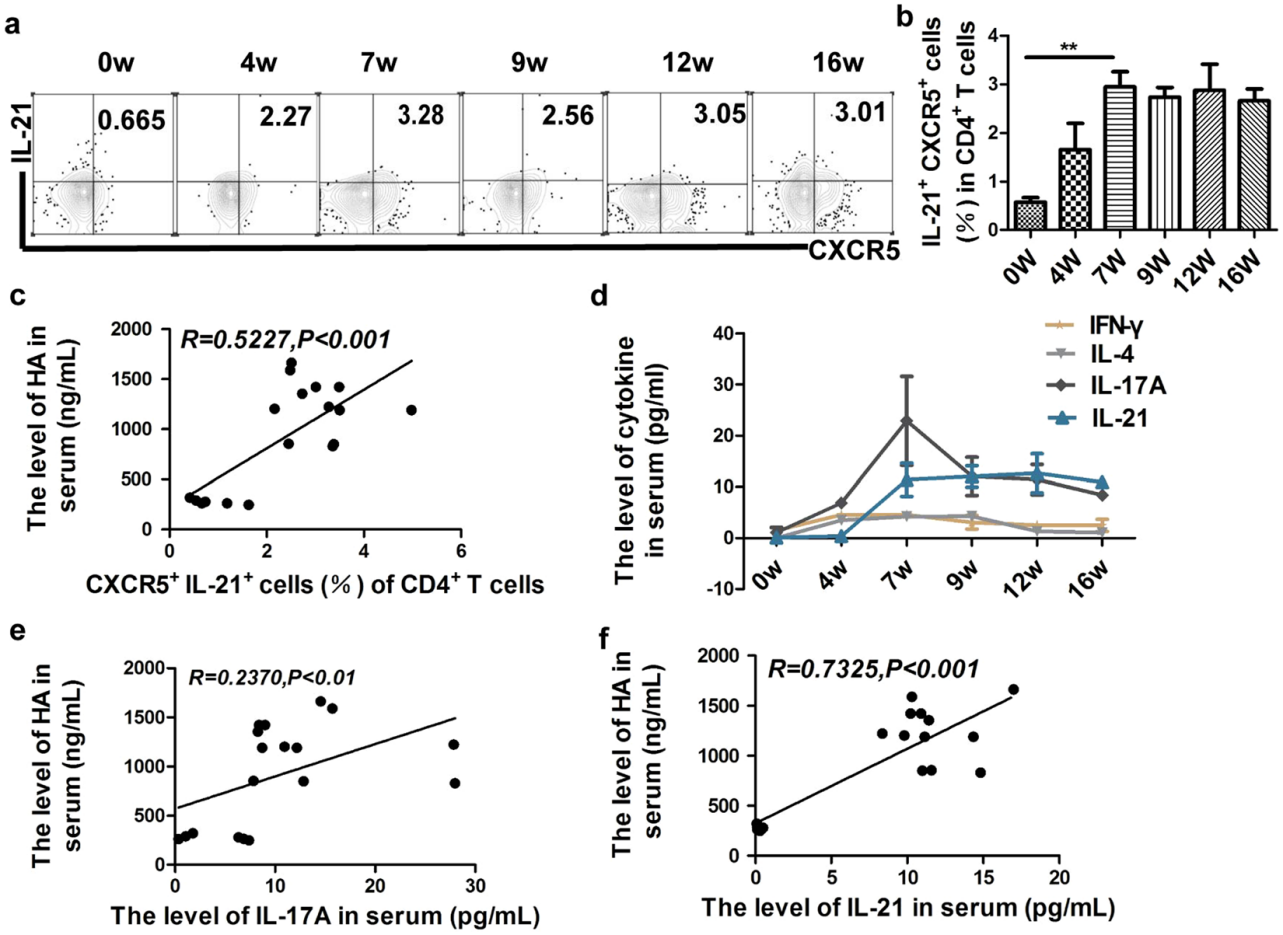
Increased Tfh-type cytokines IL-21 were positively correlated with hepatic fibrosis progression. Splenic cells were harvested from normal or infected mice (4, 7, 9, 12, 16 weeks post-infection), the splenic cells were directly stained with CD4-FITC, CXCR5-PE antibody and subsequently intracellular stained with IL-21-APC. (**a**) Numbers are indicated in the upper right of each chart which represent the percentage of IL-21^+^CXCR5^+^ cells within the CD4^+^ T cells population; (**b**) Pooled data are from two independent experiments, values are given as mean +/− SD (n = 6), **P < 0.001 (Student’s *t*-test); (**c**) The percentage of IL-21^+^CXCR5^+^ cells in CD4^+^ T cells are correlated positively with HA levels during infection of *S*. *japonicum*; (**d**) The serum samples were harvested from three replicate samples each group at different periods (0, 4, 7, 9, 12, 16 weeks), the levels of IFN-γ/IL-4/IL-17A/IL-21 in serum were determined using Luminex technology, the line chart of cytokines are expressed as the mean+/− SD (n = 3); (**e**,**f**) The level of IL-17A or IL-21 is correlated positively with the level of HA in serum in mice infected of *S*. *japonicum*.

IL-21 is a typical cytokine produced by Tfh cells and IL-21 was capable of controlling Tfh cells and Th17 cells responses^17^. Our results indicated that IL-21^+^CXCR5^+^Tfh cells were elevated at early stage and reach a plateau from acute stage to chronic stage, but still have a higher level at advanced stage (Fig. 3a and b). As expected, the levels of the proportion of IL-21^+^CXCR5^+^Tfh cells showed a positive correlation with the levels of serum HA (Fig. 3c). We also examined the kinetics of Th1, Th2, Th17 and Tfh cytokines among CD4^+^ T cells in serum during *S*. *japonicum* infection in FVB mice. Results in Fig. 3d and Supplementary Fig. 2 showed that Th1–related cytokine IFN-γ was significantly up-regulated at early stage and decreased at 9 weeks post-infection. The IL-4, IL-6, IL-10, IL-13 levels of Th2 cells continued to increase after infection during the first 9 weeks post-infection and down-regulated thereafter. Additionally, classic pro-fibrotic cytokine of TGF-β1 (Supplementary Fig. 2) and IL-17A (Fig. 3d) increased rapidly during the first seven weeks and decreased slowly thereafter. However, it is worth noting that Tfh-type cytokines IL-21 was elevated at 4 weeks post-infection and reached a plateau from week 7 to week 12 post-infection before decreasing gradually (Fig. 3d). Furthermore, the relevance of these results showed that increased levels of IL-21 or IL-17A were positively correlated with liver fibrosis progression (Fig. 3e and f). However, there was no significant correlation between IL-4, IFN-γ levels and HA levels (Supplementary Fig. 2). Our findings showed that IL-21 and IL-17A were significantly increased and associated with hepatic fibrosis progression in mice infected with *S*. *japonicum*.

### Tfh cells enriched in GC and are involved in humoral immune response by IL-21

**Figure 4:**
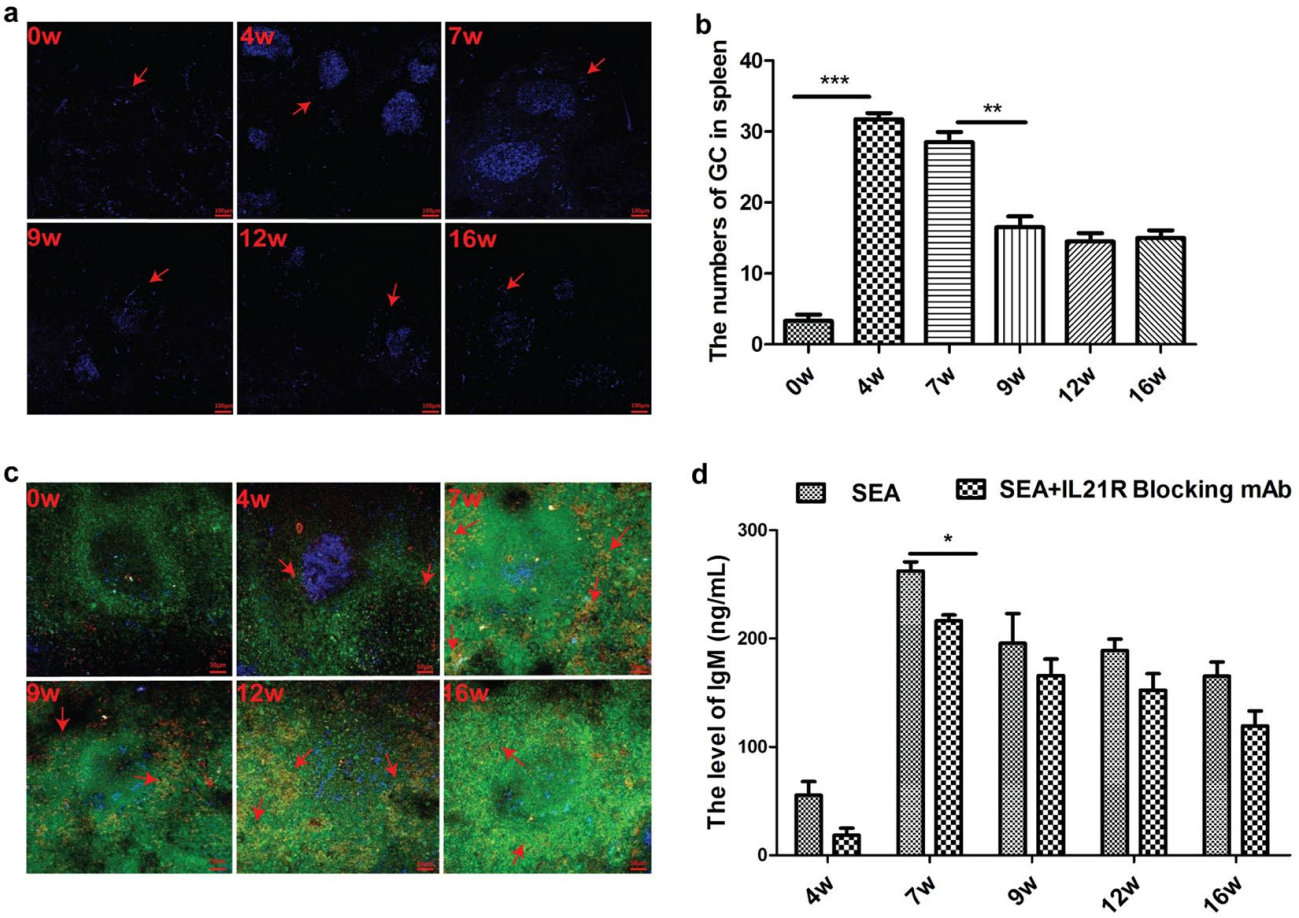
Tfh cells enriched in GC and are involved in humoral immune response. Spleen tissues were freshly harvested from normal or infected mice (4, 7, 9, 12, 16 weeks post-infection). (**a**) Paraffin-embedded section of spleen was stained PNA (blue) and GC was indicated. One representative image from two independent experiments with a total of six to eight mice is shown. Scale bar represents 100 μm; (**b**) We counted germinal center numbers of the whole spleen tissue, the graph shows mean +/− SD (n = 6–8), **P< 0.01; ***P < 0.001 (Student’s *t*-test); (**c**) Confocal images were collected from SP tissues, antibodies to CD4 (green), CXCR5 (red) and PNA (blue) and Tfh cells were indicated; (**d**) Tfh cells (CD4^+^CXCR5^+^) and B cells (CD19^+^) from normal and infected (4, 7, 9, 12, 16 weeks post-infection) spleen tissue (n = 5) were sorted by flow cytometry and co-cultured (1:1) at 37 °C for 7 days with SEA in the presence or absence of IL-21R blocking antibody or isotype-matched control antibody, IgM production was analyzed in the supernatants with a commercially available ELISA kit.

The splenic germinal center (GC) is an important structure and associated with the strength of humoral immune response. As shown in Fig. 4a and b, uninfected mice had few normal GC structure in the spleen, while a large number of normal GC were observed at 4 weeks post-infection. Although when the disease progressed from the acute to the chronic stage, the GC numbers in the spleen started to decrease gradually, the number of GC was still increased at the chronic stage compared to that before infection. Recently, some researchers reported that Tfh cells played an important role in the GC formation^9–11^. Thus, we further investigated the number variation of Tfh cells which are located at GC in mice infected with *S*. *japonicum*. Results in Fig. 4c showed that more Tfh cells migrate to GC at 4 weeks post-infection compared to that before infection and increased rapidly thereafter. So we speculated that Tfh cells may be involved in GC formation and humoral immune response in the immunopathogenesis of schistosomiasis.

Previous studies have shown that IL-21 is a key functional factor of Tfh cells, which promoted the differentiation of B cells into plasma cells or memory B cells in many diseases^22–26^. To further validate the function of Tfh in B cells during *S*. *japonicum* infection in FVB mice, we sorted Tfh cells and B cells from spleen tissue at different infection stages, and our co-culture experiment data showed that blockading IL-21 with IL-21R blocking mAb, the production of IgM in cell supernatant was lower than that the control group (Fig. 4d). This result further demonstrated that CXCR5^+^ CD4^+^ Tfh cells promoted B cells to produce more IgM immunoglobulin by up-regulating the expression of IL-21.

### Interleukin-21 cytokine drivies HSCs to secrete HA and promote liver fibrogenesis

**Figure 5:**
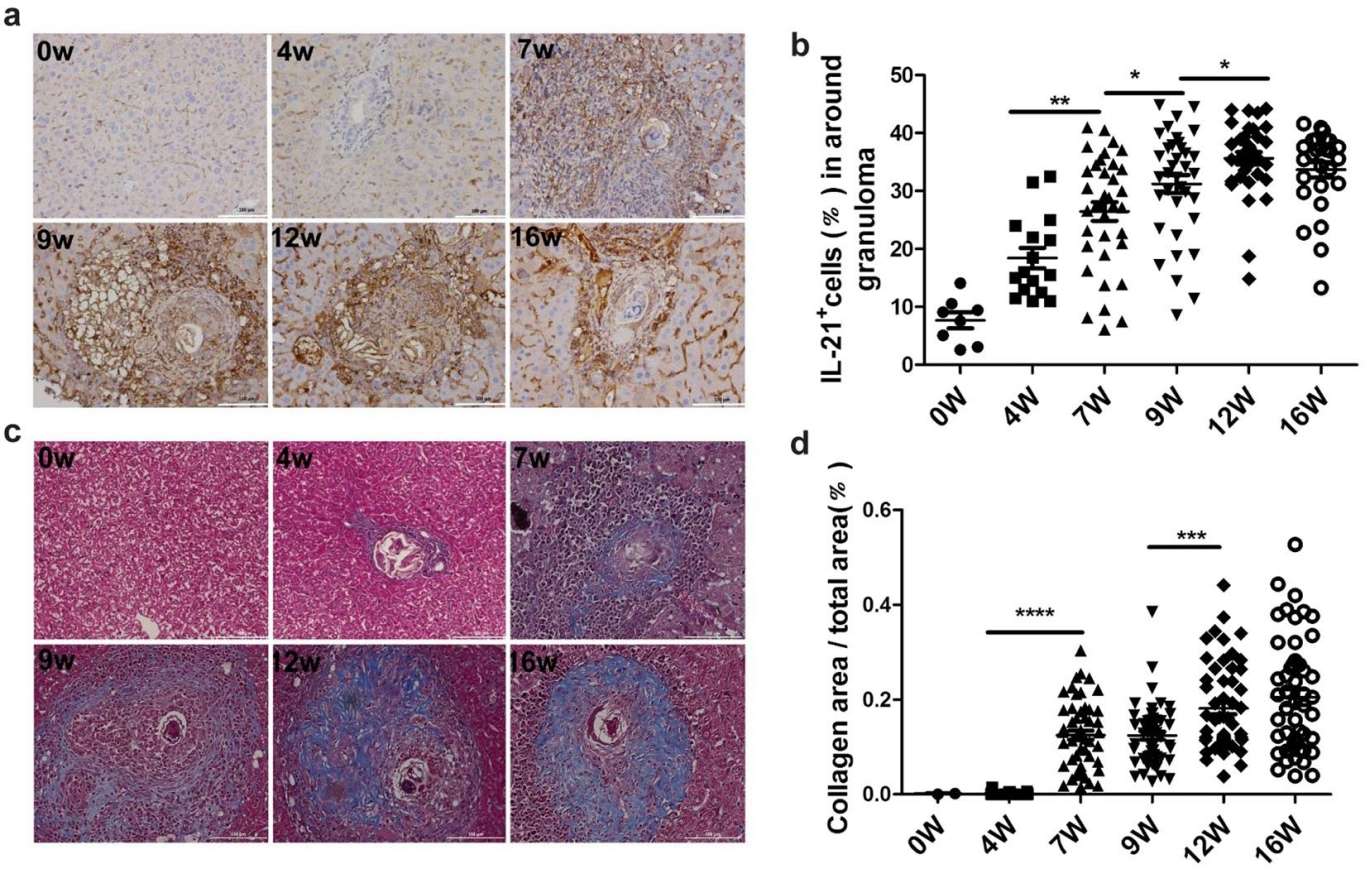
Accumulation of IL-21-producing cells in around granulomas during *S*. *japonicum* infection. Six mice of each group were sacrificed and liver tissue harvested at 0, 4, 7, 9, 12, 16 weeks after infection. (**a**) Liver sections were stained with anti-IL-21 antibody, the dark brown area in the confocal image are positive particles. Scale bar represents 100 μm; (**b**) For each Paraffin-embedded section, six fields were randomly chosen, the percentage of positive cells in around granulomas was quantified using image-pro Plus 6.0 software, the schedule shows mean +/− SD (n = 6), *P < 0.05; **P < 0.01 (Student’s *t*-test); (**c**) Masson Trichrome method determined the degree of hepatic fibrosis, confocal image shows that the blue area are collagen particles; (**d**) For each Paraffin-embedded section, nine fields were randomly chosen, the percentage of collagen area was quantified using image-pro Plus 6.0 software, pooled data shows mean +/− SD (n = 6), ***P < 0.001; ****P < 0.0001 (Student’s *t*-test).

To further elucidate the contribution of IL-21 in the development of liver fibrosis in mice infected with *S*. *japonicum*, we primarily observed whether IL-21^+^ cells were expanded in liver egg granuloma. Immunohistochemical results showed that the percentage of IL-21 positively staining cells was increased at 4 weeks post-infection compared to that before infection and reached peak at 12 weeks post-infection before decreasing gradually (Fig. 5a and b). Masson trichrome coloration method was used to observe the hyperplastic state of collagen fiber that reflected the severity of liver fibrosis. We analyzed the percentages of collagen area in around single egg granuloma and found that the dynamics of IL-21^+^ cells in liver tissue of infected mice in general consistent with that the collagen area (Fig. 5c and d), but not the granuloma size (Supplementary Fig. 3).

**Figure 6:**
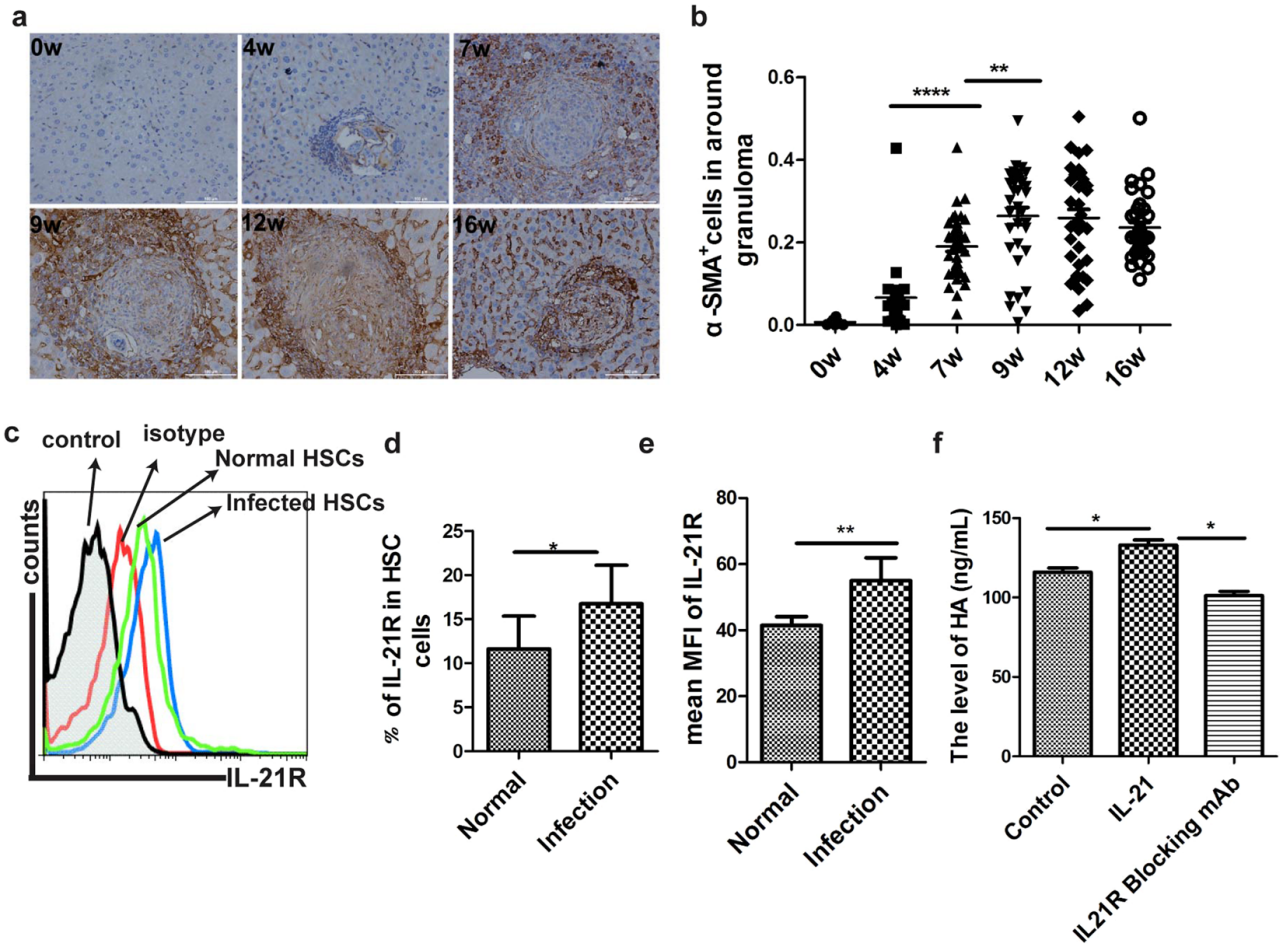
IL-21 drives HSCs secreting HA and promotes liver pathology in *S*. *japonicum*-infected mice. Six mice of each group were sacrificed and liver tissue harvested at 0, 4, 7, 9, 12, 16 weeks after infection. (**a**) The image shows the expression of a-smooth muscle actine, the dark brown indicates positive anti-a-SMA staining; (**b**) Pooled data are shown mean +/− SD (n = 6), **P < 0.01; ****P < 0.0001 (student’s *t*-test); (**c**) Flow cytometric histogram of IL-21R^+^ HSC cells from normal and infected mice; (**d**,**e**) The percentages or MFI levels of IL-21R are shown mean +/− SD from there independent experiments with a total of six to ten mice, *P < 0.05; **P < 0.01 (student’s *t*-test); (**f**) Four male mice were sacrificed at 8 weeks post-infection, freshly Tfh and HSC population were isolated from spleen and liver tissue respectively, which were co-cultured at 37 °C for 3 days dealing with IL-21R blocking mAb or IL-21 cytokine. Data were analyzed for HA concentration in the supernatant with an ELISA kit, *P < 0.05 (Student’s *t*-test).

HSCs activation is an important process to drive liver fibrogenesis, producing a large amount of collagen and resulting in liver fibrogenesis^27^. It is known that α-smooth muscle actin (α-SMA) was an important mark of HSCs activation, our results showed that the levels of α-SMA^+^ cells in liver was elevated at 7 weeks post-infection and reached at a plateau by 12 weeks post-infection (Fig. 6a and b). Importantly, this result was in consistence with the dynamics of IL-21^+^ cells in the progression of liver fibrosis. HSCs have three major characteristics, including star-like shape, spontaneous blue-green fluorescence excitated by ultraviolet of 328 nm wavelength, and specific expression of glial fibrillary acidic protein (GFAP). According to these criteria, HSCs obtained using the described protocol are more than 90% pure as assessed by immunofluorescent staining (Supplementary Fig. 4). Results in Fig. 6c showed that HSCs also expressed the IL-21R in normal mice by flow cytometry. Meanwhile, we found that the level of IL-21R in HSCs was up-regulated in mice infected with *S*. *japonicum* (Fig. 6c–e). RT-PCR assay also showed that the mRNA expression of IL-21R in HSCs was elevated in mice infected with *S*. *japonicum* compared to normal groups, but it did not reach statistical significance (Supplementary Fig. 4). Thus, we further investigated whether IL-21 cytokine could directly impact on HSCs activation and promote hepatic fibrogenesis, like IL-13 or TGF-β1. Our data showed that HSCs would produce more HA when dealing with IL-21 cytokines, while blockading IL-21 with IL-21R blocking mAb would decrease HA production compared to the control group (Fig. 6f). Taking together, these data suggested, to some extent, that Tfh cells promoting liver fibrosis is dependent on IL-21 cytokine to activate HSCs in mice infected with *S*. *japonicum*.

## Discussion

The immunity of hepatic granulomatous formation and fibrosis in schistosomiasis has attracted extensive research interest. It is well known that the irreversible liver damage was predominantly caused by the humoral and cellular immune response the schistosome egg antigens^1,28^. Thus, to investigate the exact immune regulatory mechanism of hepatic egg granuloma formation and subsequent fibrosis is dispensable for the treatment of patients with schistosomiasis. In this study, we demonstrated the key role of IL-21 producing cells to promote liver pathology, particularly in liver fibrogenesis in murine schistosomiasis.

T follicular helper (Tfh) cells is a new subset of CD4^+^ T cells, which have a distinct gene expression profile and independently of Th1, Th2, Th17 cell lineages^9^. Some evidence supported that the number of Tfh cells was increased in various infected diseases such as immune-active chronic hepatitis B and hepatitis C virus^5,6^. According to the latest report, Tfh cells were expanded and promoted liver granulomatous formation in mice infected with *S*. *japonicum* ^8^. However, whether Tfh cells, as the Th17 cells do, contribute to the subsequent liver fibrosis is still unknown. In this study, our data suggested that Tfh proliferation was positively associated with liver fibrogenesis. Thus, we speculated that Tfh cells not only played a key role in granulomatous formation, but also involved in subsequent fibrosis in schistosomiasis. However, the exact mechanism and function of Tfh cells in granulomatous formation and subsequent fibrosis in schistosomiasis remains to be further explored.

Studying the specific phenotype and basic immunologic characteristics of Tfh cells is necessary for further elucidating and understanding the development and functions of Tfh in various infectious diseases. Up to now, that Tfh differentiation and generation was mainly controlled by the transcription factors Bcl-6 was widely accepted^19,29,30^. Using the mice model of schistosomiasis, the present study also indicated that Bcl-6^+^CD4^+^ cells would express higher CXCR5 of Tfh marker molecules than Bcl-6^−^CD4^+^ cell, the ability of Bcl-6 to up-regulate the expression of CXCR5 was strengthened in mice following *S*. *japonicum* infection. Furthermore, we also found that Bcl-6 instructed Tfh development to commence early in murine schistosomiasis. Extensive evidence suggested that naive CD4^+^ T helper (Th) cells recognize antigens presented by antigen-presenting cells (APCs) to differentiate into Tfh cells under specific co-stimulatory molecules and cytokines milie (such as ICOS/ICOSL, CD40/CD40l, IL-21 and IL-6), which is similar to other CD4^+^ T cell populations (Th1, Th2, Th17, and Tregs) differentiation^9,19,31–35^. Furthermore, Tfh activation and function were closely related with high expression of ICOS, PD-1. In this study, our findings indicated that ICOS^high^, PD-1^high^Tfh cells were not only associated with severe hepatic granulomatous inflammation and subsequent fibrosis, but also the proportion of Tfh in CD4^+^ T cells increasing. IL-21, one important functional factor and secreted mainly by Tfh cells,has been implicated in inducing Th2, Th17 and Tfh cells differentiation and responses^17,34,36,37^. HA, the polysaccharide substance, is the principal component of proteoglycan for extracellular matrix (ECM) and degraded in the liver endothelial cells. Serum hyaluronic acid is a comprehensive marker to assess severity of liver disease in schistosomiasis^20,21,38,39^. The data presented here indicated that the proportion of IL-21 producing Tfh cells also have a striking correlation with HA levels. Thus, it might reveal that Tfh-type cytokine IL-21 contributed to Tfh response in the development of immunopathology in murine schistosomiasis. In summary, all of these results suggested that the dynamics and effects of Tfh cells in immunopathgenesis of murine schistosomiasis provide an important immunological basis for the involvement of Tfh development and function.

It is known that CD4^+^ T cells population, including Th1, Th2, Th17 and Treg cells characterized by related typical cytokines were involved in the liver pathology in mice and human schistosomiasis^40–43^. An early study of our group showed that IL-17A contributes to the hepatic granulomatous inflammation and subsequent fibrosis^40^. The data presented here of Luminex analysis displayed markedly correlation between the level of IL-21 or IL-17A in serum and disease severity of schistosomiasis. However, there was no tendency of correlation between the level of IL-4 and the level of HA in mouse schistosomiasis, it possibly only contributes liver granulomatous formation, but not liver fibrogenesis. In summary, these findings might suggest thatIL-21 and IL-17A contribute to driving fibrogenesis in schistosomiasis.

GCs is a specialized immunity structure, follicular Tfh cells play a predominant role in the strength of humoral immune response by providing help to B cells for high-affinity antibody production as well as the formation of GC^9–16^. In this study, our result showed that *S*. *japonicum* infection drives normal GC formation. Our findings further indicated that with the development of fibrosis, more Tfh cells were migrated into GC and promote B cell humoral immune response. Using the IL-21^−/−^ mouse model, Vogelzang *et al*.^34^ found that IL-21 was necessary for the formation of GC. However, the central role of IL-21 in GC formation exhibited its impact on Tfh cells generation rather than on B cells. Furthermore, studies showed that IL-21 cytokines of Tfh cells contributed to the development of pathology by providing help to B cells to produce high level of immunoglobulin, which was associated with the long-term humoral immune response in autoimmune diseases^23,24,29,44^. However, neither the significance nor the role of IL-21 producing Tfh cell in the mediation of humoral immune response, to the exacerbation of hepatic fibrosis in mice infected with *S*. *japonicum* have been validated. Consistent with previous studies, our co-culture experiment data also suggested that Tfh cells had elevated IgM antibodies production, which, to some extent is dependent on IL-21 in schistosomiasis. Taken together, Tfh cells are involved in the development of inflammatory pathology by IL-21 driving GC response in schistosomiasis.

A previous study indicated that the circulating Tfh cells in peripheral blood originated from GC-Tfh cells and had a nearly uniform phenotype^45^. Furthermore, peripheral circulating Tfh cells were recruited or migrated to the liver and promoted granuloma formation^8^. However, little is known of the exact mechanism of Tfh cells in liver granuloma formation and subsequent fibrosis in schistosomiasis. Some studies have focused on the contribution of IL-21 to inflammatory disease such as autoimmune hepatitis, rheumatoid arthritis^46–48^. In the present study, the tendency of IL-21^+^ cells in liver was consistent with IL-21 changes in serum during *S*. *japonicum* infection. Thus, we speculated that IL-21^+^ cells in liver might play a critical role in the development of fibrosis in mice infected with *S*. *japonicum*. HSCs is located in liver disse gaps and next to hepatocyte and liver sinusoidal endothelial cell, displaying a star-like morphology and perinuclear vitamin A-storing lipid vesicles^49^. HSCs have the characteristics of specific expression of GFAP. It is known that the activation of HSCs was a key link to promote liver fibrogenesis in fibrosis disease^27^. The expression of IL-21R was found on some immune cells (T cells, B cells, macrophages) and nonimmune cells (epithelial, myofibroblast)^50^. Recent study also suggested that IL-21R was observed on LX-2 cell lines (immortalized human HSCs), and the expression of α-SMA on LX-2 cells up-regulated significantly by adding IL-21 cytokines, which is an important mark of HSCs activation^50^. Our data suggested that HSCs also express the IL-21R in normal mice and the mice infected with *S*. *japonicum* displaying markedly elevated expression level of IL-21R on HSCs compared to normal mice. More importantly, blockading IL-21 cytokines could inhibit the secretion of HA produced from HSCs, while HSCs drive more HA production by adding IL-21. These findings suggested that IL-21 could promote HSCs activation directly and result in hepatic fibrosis. In conclusion, we proposed that IL-21 would act as a potential immune therapeutic target for anti-fibrosis in schistosomiasis. However, whether IL-21, as other profibrogenic cytokines such as TGF-β1 and IL-13 characterized by Smad signaling pathway, plays an important role in the development of hepatic fibrosis is worthy of further research.

In summary, our findings indicated that Tfh cells could drive hepatic fibrogenesis in addition to Th17 cells in schistosomiasis. Importantly, IL-21 was identified as a promotor to promote pathology by driving GC formation and activating HSCs. Our study contributes to better understand the immunopathogenesis of schistosomiasis and indicated that the IL-21 might be a potential therapeutic targeting factor for liver fibrosis in schistosomiasis.

## Methods

### Ethics statement

The animal experiments using mice were preformed in strict accordance with the Regulations for the Administration of Affairs Concerning Experiments Animals (1988.11.1), and humanely handled. All animal procedures were approved by the Institutional Animals Care and Use Committee (IACUC) of Soochow University for the use of laboratory animals (Permit Number: 2007–13).

### Mice, parasites and infection

Female FVB mice (6–8 weeks old) were purchased from the Center of Comparative Medicine of Yangzhou University (Yangzhou, China). Animals were kept under specific pathogen-free condition at the Laboratory Animal Research Facility of Soochow University (Suzhou, China). Mice were infected with 15 ± 1 *S*. *japonicum* cercariae (Chinese mainland strain) hatched from infected O*ncomelaniahupensis* snails purchased from Jiangsu Institute for Parasitic Control (Wuxi, China). SEA was obtained from Jiangsu Institute for Parasitic Control (Wuxi, China).

### Flow cytometry

Single-cell suspensions were prepared from fresh spleen tissue and followed by lysis of red blood cell (RBC) using ACK lysis buffer. For surface staining, 1 × 10^6^ splencytes per 100 μl were incubated for 30 min at 4 °C with the following fluorescently labeled conjugated mAbs: CD4-FITC (eBioscience, San Diego, CA), CXCR5-PE (eBioscience), CXCR5-APC (eBioscience), ICOS-PE-Cy5.5 (eBioscience), OX40-APC (eBioscience), CD40L-APC (eBioscience), PD-1-APC (eBioscience), CD19-APC (eBioscience), IL-21R-PE (BioLegend). After staining of surface markers, the cells were permeabilized with fixation/permeabilization solution (eBioscience) and subsequently intracellularly stained with Bcl-6-PE (BD Pharmingrn, San Jose, CA), the cells were then wash twice using permeabilization buffer, and eventually fixed with 4% paraformaldehyde in PBS. For APC-conjugated anti-IL-21(BD Pharmingrn), spleen cells were stimulated with PMA and Ionomycin for five hours and subsequently intracellularly stained, all procedures were in accordance with Bcl-6 staining method. In addition, Tfh (CD4^+^CXCR5^+^) subsets and B (CD19^+^) subsets were sorted with a FACS Aria cell sorter (BD Bioscience).

### Serum experiments

Serum HA titers of normal or infected mice (4, 7, 9, 12, 16 weeks post-infection) were measured by Hyaluronan Quantikine ELISA Kit (R&D Systems, Minneapolis, MN USA) according to the manufacturer’s protocol. The levels of IFN-γ, IL-4, IL-17A, IL-21 of Tfh in normal or infected mice (4, 7, 9, 12, 16 weeks post-infection) were determined using ProcartaPlex Mouse Immunoassay Kit (eBioscience).

### Quantitative real-time PCR

Total RNA was extracted from Tfh cells and HSCs with QIAGEN RNeasy kit and reverse transcribed into cDNA with PrimeScript RT reagent Kit (TaKaRa, Beijing, CN). We determined the mRNA expression of ICOS, PD-1, Bcl-6 and IL-21R by the ABI PRISM 7500 Sequence Detection System (Applied Biosystems) with SYBR Green Real-time PCR Kit (Applied Biosystems). The sequence primers of Tfh phenotype molecules were showed in S1 Table.

### B-Tfh Co-Culture assay

Sorted spleenic Tfh cells were co-culture with autologous CD19^+^B cells in the presence of SEA (20 µg/ml) in 96-well U-bottom plates. In blocking experiments, endogenous IL-21 was neutralized by IL-21R blocking mAb (5 µg/ml, eBioscience) or an isotype-matched control. Secretion of total IgM was determined by ELISA (Abcam) after 7 days in culuture.

### HSCs isolation, identification and culture

Mice (9 weeks after infection) were anaesthetized with Chloral Hydrate and sterilized with 70% alcohol. The abdominal cavity was opened with surgical scissors. Preheating perfusion buffer 1 (RPMI 1640, Sigma Corporation, St. Louis, USA) was pumped into the liver via the hepatic portal vein and drained via the Vena cava inferior with a peristaltic pump. After the blood was washed clean, preheating perfusion buffer 2 (0.04% Collagenase I (Gibco, Grand Island, NY) in RPMI 1640, adjusted to a pH 7.3–7.4 using 10 N NaOH, sterile filtered, and kept at 37 °C until use) was injected into liver for 6 min with a peristaltic pump at a flow rate of 15 ml/min. The liver was grinded and digested at 37 °C for 30 min with 20 ml oscillating digestive buffer (0.08% pronase E (Roche), 0.08% collagenase I, and 5U/ml DNase I (Sigma) in RPMI 1640, adjusted to pH 7.3–7.4 using 10 N NaOH, sterile filtered, and kept at 37 °C until use). The digestion was terminated immediately with 20 ml RPMI 1640 and filtrated using 70 μm nylon gaze (BD Bioscience). The liver cell suspension was further centrifuged at 4 °C and 600 g for 6 min, washed twice with RPMI 1640 buffer. The last step was density gradient centrifugation with Optiprep (Axis-Shield PoC AS), for 20 min at 3000 g without brake. HSCs obtained using the described protocol and fixed using 4% paraformaldehyde for 60 min. 10% normal goat serum (Boster, Wuhan, China) was used to inhibit unspecific binding. GFAP staining was done for 12 hours at 4 °C using rabbit anti-mouse GFAP polyclonal antibody (Boster, Wuhan, China). Secondary antibody incubation followed by biotin-labeled goat anti-rabbit IgG for 30 min at 37 °C and further incubated with SABC-Cy3 for 30 min at 37 °C. Finally, slides were mounted using Vectashield Mounting medium (Millipore). Ten random digital images of each slides were captured using microscope (OLYMPUS) and the ratio of GFAP^+^ cell is calculated. The mean was the purity of HSCs. The obtained objective cells were then washed twice using RPMI 1640, and 1 × 10^5^ cells were inoculated in 96-well culture plate (Costar, Cambridge, MA), changing liquid for 24 hours. The sorted Tfh cells (2 × 10^5^ cells, 8 weeks post-infection) were seeded into culture plate when all HSCs completely adhere and deformed at 72 hours, continued to culture with IL-21 cytokines (Peprotech, Rocky Hill, NJ, USA) and IL-21R blocking mAb (eBioscience) for 72 hours at 37 °C in 5% CO2 condition before the supernatants were harvested.

### Immunohistochemistry

Mice were sacrificed and liver tissues were harvested at 0, 4, 7, 9, 12, 16 weeks and fixed in 10% neutral buffered formalin. Liver sections (4 μm) were treated with 0.3% H2O2 and 10% normal goat serum (Boster, Wuhan, China) to block endogenous peroxidases and non-specific binding. The slides were incubated with anti-alpha smooth muscle actin (Abcam) and anti-IL-21 (Abcam) for 1 hours and further incubated with the secondary antibodies (ChemMate Envision^+^/HRP/DAB, rabbit/mouse detection kit, Gene-tech, Shanghai, China). Six random digital images of each mouse were captured using a light microscope (OLYMPUS) and the dark brown were positively staining cells which were analyzed with Image-Pro Plus 6.0 software.

### Immunofluorescence histology

Spleen was freshly harvested from normal or infected mice (4, 7, 9, 12, 16 weeks post-infection) and fixed in 10% neutral buffered formalin. Paraffin-embedded sections (4 μm) of spleen were cut using Lecia CM1900 cryostat. Briefly, after incubation with primary antibodies, CD4^+^ T cells, B cells, GCs were stained with anti-CD4 (Santa Cruz), anti-CXCR5 (Millipore), PNA (Vector Laboratories) respectively. Secondary antibodies incubation followed by Alex Fluor 488, Alex Flour 647, Alex Flour 405 (Invitrogen, Carlsbad, CA). All images were captured at 200× magnification using a ziss Axiovert 200 M microscope and analyzed using Axiovision software.

### Histopathology

Mice were sacrificed and liver tissues were harvested at 0, 4, 7, 9, 12, 16 weeks and fixed in 10% neutral buffered formalin. Liver sections (4 μm) were stained with Hematoxylineosin or Masson Trichrome. To observe the area of granulomas, for each HE-stained slide, we randomly captured 15 images of granulomas around single eggs and measured with image-pro Plus 6.0. However, each liver section has little granulomas at early stage in mice infected with *S*. *japonicum*, so all granulomas images were collected and analyzed. To assess the degree of hepatic fibrosis, for each mouse, six images were collected randomly, Masson Trichrome stained sections were collagen positive particles, the ratio of collagen area was counted with Image-pro Plus 6.0. All digital images were captured by a light microscope (OLYMPUS).

### Statistical analysis

All data were analyzed using Prism 6.0 software (GraphPad Software, Inc, San Diego, CA). The significance of the difference between all the groups which have different periods (0, 4, 7, 9, 12, 16 weeks post-infection) was compared by unpaired, two-way Student’s test. The difference between the treatment groups was analysed using paired t-test. Spearman’s correlation was used to analyze the potential relationship between two variants groups. *P* < 0.05 was considered as statistically significant.

## Electronic supplementary material

Supplementary Information
