# Supplementary material for: Up-regulation of Interleukin-21 Contributes to Liver Pathology of Schistosomiasis by Driving GC Immune Responses and Activating HSCs in Mice
Source: Sci Rep. 2017 Nov 30;7:16682. doi: 10.1038/s41598-017-16783-7 (PMC5709429; doi:10.1038/s41598-017-16783-7)
Supplement: Supplementary file 1 — Supplementary Information [file 41598_2017_16783_MOESM1_ESM.doc]

**Up-regulation of Interleukin-21 Contributes to Liver Pathology of Schistosomiasis by Driving GC Immune Responses and** **Activating HSCs in Mice**

Yanyan Wang^1¶^, Cai Lin^1¶^, Yun Cao^1^, Zhongliang Duan^1^, Zhixun Guan^1^, Jing Xu^1^, Xing-Quan Zhu^2^, Chaoming Xia^1*^

**Supplementary figure legends**

**Supplementary Figure 1. The correlation between the percentage of PD-1^+^Tfh cells and the level of HA in serum during  *S.japonicum* infection.** (a)The serum samples were harvested from three replicate sample each group at different periods (0, 4, 7, 9, 12, 16 weeks), the level of HA in serum were determined using ELISA method;(b)(c) the percentage of CXCR5^+^ Tfh cells and PD-1^high^ Tfh cells were correlated positively with the level of HA in serum;(d) PD-1^high^ Tfh cells increasing was correlated positively with Tfh cells proliferation.

**Supplementary Figure 2.** **Cytokines production by Th cells in serum during *S.japonicum* infection.** The serum samples were harvested from three replicate sample each group at different periods (0, 4, 7, 9, 12, 16 weeks), (a)(b)(c)(d) The levels of IL-6, IL-10, IL-13, TGF-β1 in serum were determined using Luminex technology, the line chart of cytokines are expressed as the mean +/− SD (n=5).(e)(f) The correlation between the level of IL-4 or IFN-γ and the level of HA in serum in mice infected of *S.japonicum*.

**Supplementary Figure 3. Pathological structure was observed by Hematoxylineosin staining during *S.japonicum* infection**. Six mice of each group were sacrificed and harvested liver tissue at 0, 4, 7, 9, 12, 16 weeks. (a) Hematoxylineosin staining to observe pathological structure; (b) For each section, the size of 15 granulomas around single eggs were measured with image-pro Plus 6.0 software. Values are given as mean +/− SD (n=6), *, p<0.05; ****, P<0.0001(Student’s t-test).

**Supplementary Figure 4. Characteristic morphology and**  **identification of hepatic stellate cells**. HSCs were isolated from mouse livers using the protocol provided. (a) HSCs exhibit spontaneous blue-green fluorescence excitated by ultraviolet of 328 nm wavelength on day 1 in *vitro* culture; (b) HSCs express GFAP on day 5 in *vitro* culture;(c) HSCs were sorted in liver from normal and infected mice and analyzed the mRNA expression level of IL-21R using 2^-△△CT^ method and the housekeeping genes was β-actin, data are shown mean +/− SD (n=4-8), NS ,p>0.05 (student’s t-test).

**Supplementary figure 1.**


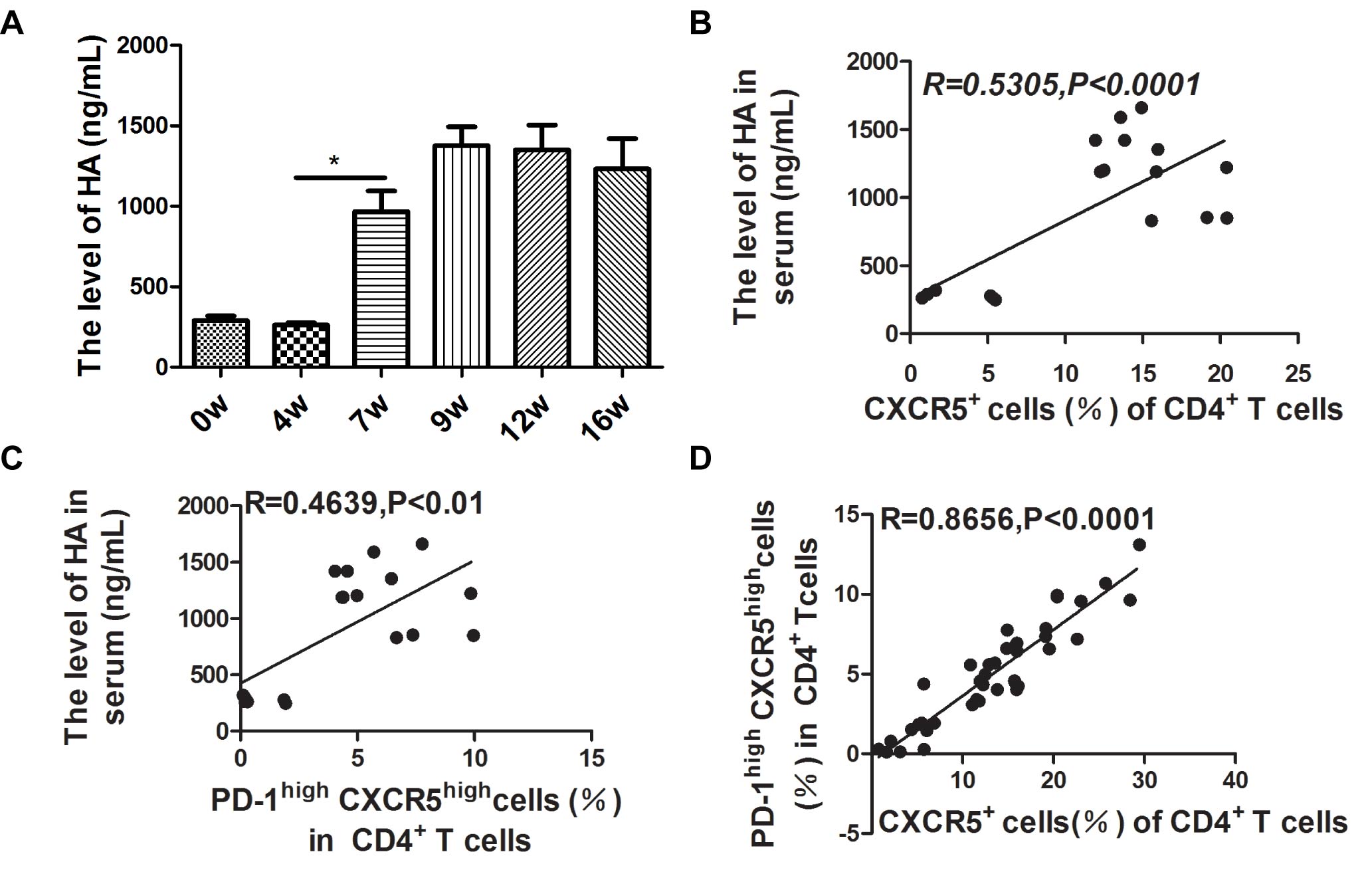


**Supplementary figure 2.**


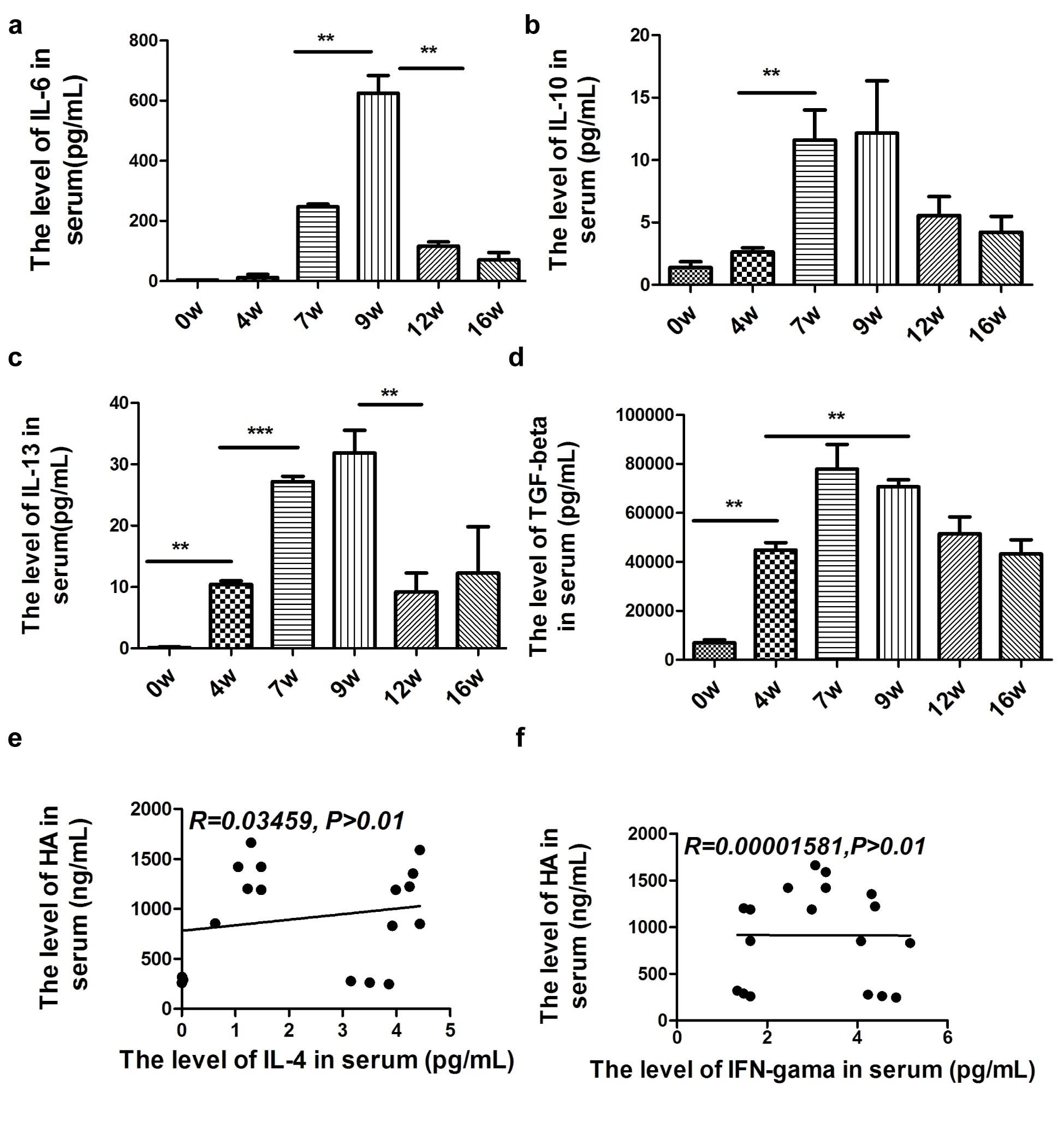


**Supplementary figure 3.**


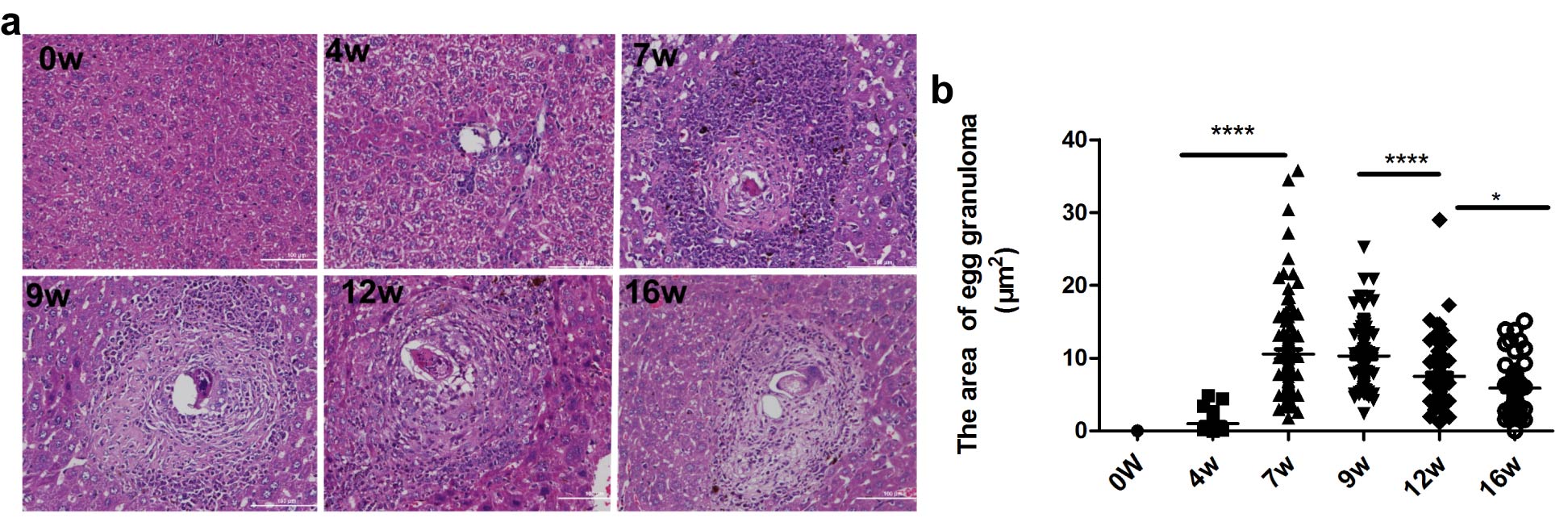


**Supplementary figure 4.**


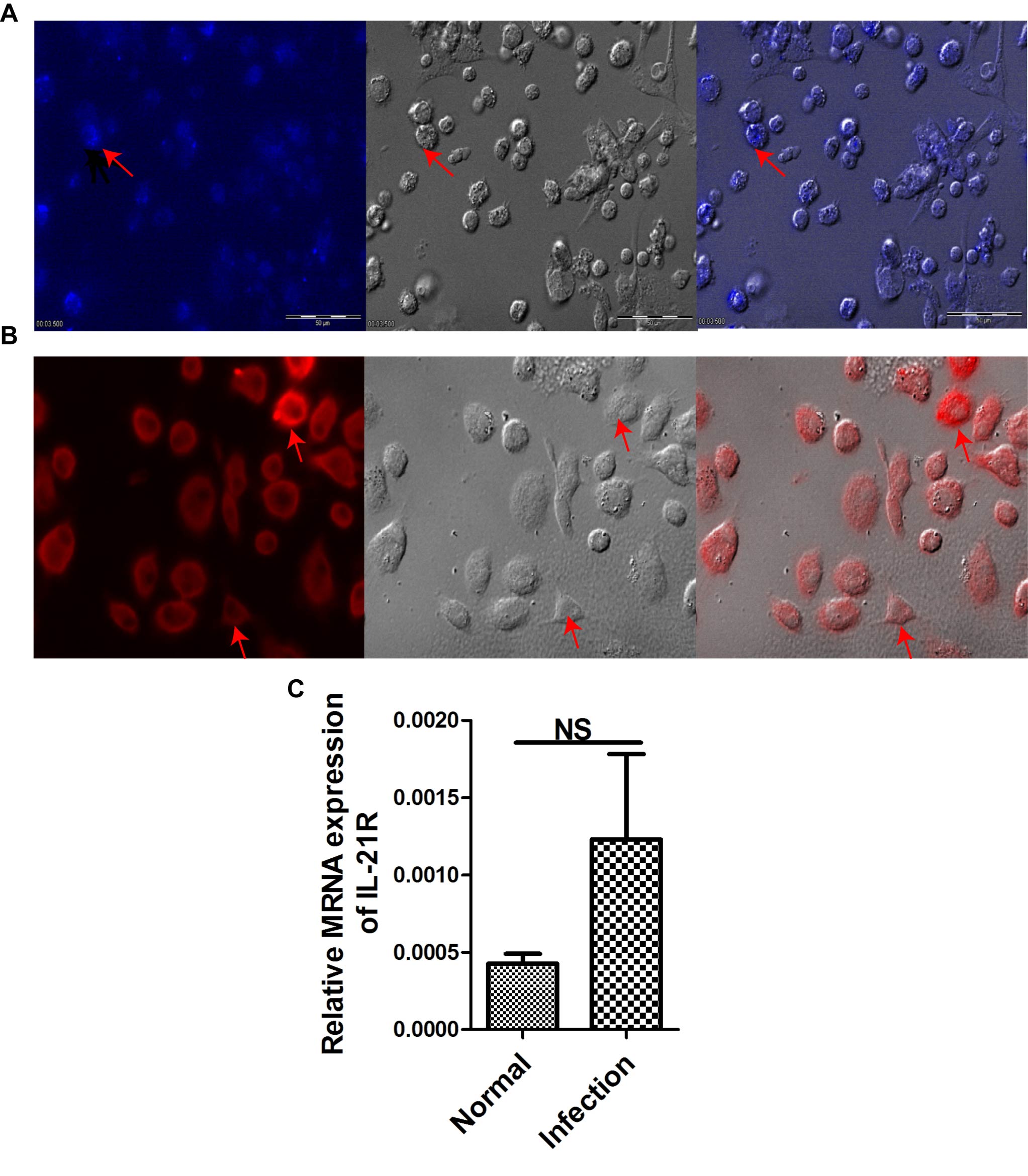


**Supplementary Table 1.** **The sequence primers of Tfh phenotype molecular were used.**
